# The Importance of Professional Discourse for the Continual Advancement of Practice Standards: The RBT® as a Case in Point

**DOI:** 10.1007/s10803-020-04631-z

**Published:** 2020-08-06

**Authors:** Justin B. Leaf, Ronald Leaf, John McEachin, Andy Bondy, Joseph H. Cihon, Ronnie Detrich, John Eshleman, Julia L. Ferguson, Richard M. Foxx, B. J. Freeman, Peter Gerhardt, Sigrid S. Glenn, Megan Miller, Christine M. Milne, Toby Mountjoy, Tracee Parker, Joshua Pritchard, Robert K. Ross, Melissa S. Saunders, Todd Streff

**Affiliations:** 1 Autism Partnership Foundation, BCBA-D. 200 Marina Drive, Seal Beach, CA 90740 USA; 2 Pyramid Educational Consultants, Inc, New Castle, DE USA; 3 Detrich and Associates, Logan, UT USA; 4 The Chicago School of Professional Psychology, Chicago, IL USA; 5 Penn State University, Pennsylvania, PA USA; 6 UCLA School of Medicine, Los Angeles, CA USA; 7 The Epic Program, Paramus, NJ USA; 8 University of North Texas, Denton, TX USA; 9 Acron Health, Coral Gables, FL USA; 10 Autism Partnership Hong Kong, Quarry Bay, Hong Kong; 11 Factari, Central Florida, FL USA; 12 Beacon ABA Services, Milford, MA USA; 13 Creative Interventions, Windsor, CT USA; 14 Streff Behavior Consulting, Foristell, MO USA

**Keywords:** Applied behavior analysis, Autism, Behavior Analysis Certification Board®, Registered Behavior Technician™

## Abstract

The Behavior Analyst Certification Board (BACB®) created a third level of certification, the Registered Behavior Technician™ (RBT®) in 2014. The RBT® was created based upon the requests of stakeholders who wanted to credential those individuals who make direct contact with clients under the supervision of a Board Certified Behavior Analyst®. There has been tremendous growth in the number of RBTs® with over 60,000 individuals certified to date. The BACB® recently sent out a newsletter outlining changes to the RBT® certification, including the processes of training, supervising, and becoming an RBT®. These changes represent a number of potential concerns. The purpose of this paper is to highlight these concerns and to propose solutions to improve the RBT® certification.

Early and Intensive Behavioral Intervention (EIBI) has been shown to dramatically change the developmental trajectory of children diagnosed with ASD (Eldevik et al. 2012; Leaf et al. 2011; Lovaas 1987; McEachin et al. 1993). As such, there has been an increasing demand for qualified professionals providing EIBI. This has been paralleled with efforts to ensure consumers, or those contacting EIBI, are protected from potential harm (Behavior Analyst Certification Board® n.d.). One of these efforts was the creation of the Behavior Analysis Certification Board® (BACB®) to help ensure minimal standards across practitioners and, as a result, protect consumers (Behavior Analyst Certification Board® n.d). There has been tremendous growth in the number of individuals who are now certified behavior analysts.^1^ For example, in 1999 there were only 28 Board Certified Behavior Analysts (BCBAs®) and two Board Certified Assistant Behavior Analysts (BCaBAs®) worldwide (Behavior Analyst Certification Board® n.d.), increasing to 36,106 BCBAs® and 3734 BCaBAs® by October, 2019 (Behavior Analyst Certification Board® n.d.).

In 2014, the Behavior Analyst Certification Board (BACB®) developed a third certification level, the Registered Behavior Technician (RBT®).^2^ Carr et al. (2017) reported that the RBT® credential was created “in response to various stakeholders (e.g., funders, legislators) who sought a credential for the individuals who actually make direct contact with clients” (p. 164). Specifically, an individual with an RBT® certification is a person who: (a) is at least 18 years old, (b) has a high school diploma or national equivalent, (c) passes a criminal background check, (d) completes a 40 h training based upon the task list the BACB® has created, (e) passes a direct observation competency assessment, (f) passes a written exam, and (g) pays all fees (Carr and Nosik 2017). Additionally, RBTs® are required to practice under the supervision of a BCBA®. It should also be noted that while the RBT® credential is not autism specific, based on the large number of BCBAs® that work in autism related fields (Behavior Analyst Certification Board n.d) it is likely that the majority of RBTs® do as well.

The RBT® credential has been widely adopted as indicated by the growth in the number of RBTs® (i.e., over 66,000 RBTs® as of October 1, 2019; Behavior Analyst Certification Board n.d.) and that direct work by RBTs® can now be reimbursed by third party payers and funding streams (e.g., Tricare; US Department of Defense 2019). As the RBT® credential has become widespread, the BACB® has made changes to various aspects of the credentialing process. Many of these changes have been notable improvements (Nosik 2018) whereas others have raised some concerns.

Given the continual growth in the field of behavior analysis and the ascent of the RBT®, it is vital for professionals to continue to critically evaluate the parameters, process, and requirements for RBT® certification. These evaluations should highlight the changes that produce positive outcomes, those that could worsen conditions, and offer potential alternatives and solutions. The evaluations should also be ongoing to establish a feedback loop for continual revision and improvement to our certification processes and standards regarding the RBT® and certification practices in general.

The 20 authors of the current manuscript are concerned with the current state of training and the RBT® certification. We have come to a consensus that the RBT® certification is shifting the training of interventionists away from implementing interventions grounded in the science of ABA. Moreover, we are committed to ensuring quality behavioral intervention is available for individuals diagnosed with ASD, individuals diagnosed with ASD live high quality lives, and that setting standards for behavior analysts does not become a race to the bottom. Failure to achieve these commitments could result in behavior analysis becoming less accepted and jeopardizing funding for individuals diagnosed with ASD.

The purpose of this paper is not to rehash old concerns or to continue fueling potentially fundamental disagreements between some of the authors and the members of the BACB®. Rather, we hope to continue the discussion started by Leaf, Leaf et al. (2017) and Carr et al. (2017) by orienting all ABA practitioners to our shared responsibility to continuously asking challenging questions that will result in direct, ongoing, and critical evaluation of certification practices as well as improved services for the populations served by applied behavior analysts. We will highlight concerns with the current RBT® certification and provide potential solutions. Although we outline concerns regarding behavior analysts, and specifically the RBT®, they are not intended solely for behavior analysts but rather, for anyone with a connection to an individual diagnosed with ASD (e.g., parent, friend, sibling, professional) regardless of their role or discipline.

## Advancements

The BACB® has made changes to improve the RBT® training and process which should be commended. First, shaping and how to use token economies were added to the 2nd edition of the competency task list. Shaping was a critical addition since it has always been an important part of behavioral intervention for individuals diagnosed with ASD (Galbicka 1994). Similarly, token economies (Ayllon and Azrin 1968; Ghezzi et al. 2003) have long been implemented successfully in the settings in which many RBTs® commonly work. Given the documented importance of these skill sets, their addition to the task list was important and welcomed.

A second notable change was expanding the scope of persons who can provide training and conduct the competency assessment for RBT® candidates. This change allows assistant trainers to be persons other than a BCBA® or BCaBA®. For instance, a noncertified professional trained in the principles of behavior analysis with years of experience but has elected not to become certified can assist in the RBT® training process. This change could help increase the number of professionals available to provide training as well as enhance the quality of training.

Finally, the BACB® has mandated that RBT® candidates display at least three of ten tasks (e.g., discrete trial teaching, chaining, extinction) with *actual clients* during the competency assessment, as opposed to role-playing with a pseudo client. This is a step toward creating a testing environment that more closely aligns with the environment in which the RBT® candidate will likely use the targeted skills (i.e., implementing an intervention with a client).

## Concerns

Despite the aforementioned advancements, several concerns related to ensuring individuals diagnosed with ASD receive high quality behavioral intervention and protecting the field of behavior analysis remain. These concerns are highlighted and discussed below.

### #1: Who can be Assistant Trainers and Assistant Assessors

Perhaps one of the biggest concerns is related to the addition of the assistant trainer, defined as an individual “who has demonstrated, through direct observation, proficiency in the material being delivered. This individual does not need to be certified by the BACB, but the trainer will be professionally and ethically accountable for all of the assistant trainer’s activities” (Behavior Analyst Certification Board 2018b, p. 4). While this change could be positive in many aspects (as discussed previously), how the assistant trainer and assessor are defined could present some additional challenges.

First, without a clear definition of “proficiency in the material being delivered” (Behavior Analyst Certification Board 2018b, p. 4) each BCBA® and/or BCaBA® determines what constitutes proficiency. It is not unreasonable to presume that new assistant trainers receiving training only by another assistant trainer could present a substantial risk of drift from the originally intended standard. Second, the assistant trainer or assessor can be an RBT®. When RBT® certification began, only a BCBA® or BCaBA® could provide supervision to an RBT® (Behavior Analyst Certification Board 2013); thus, only credentialed professionals provided supervision. Based upon the current definition, it is now possible, and we fear it is likely, that an individual could receive the RBT® credential and become an assistant trainer. This creates a situation whereby someone has met a minimal training requirement provides the same training for others. This possibility represents a severe degradation of our training standards and a move in the wrong direction.

### #2: Requiring Only a Minimum of 40 Hours of Training

The requirement of 40 h of training to become eligible for the RBT® competency assessment and examination remains deeply concerning and is inconsistent with the research on training staff to develop competency on the items included on the RBT® task list (Leaf, Leaf et al. 2017). As an example, recent literature reviews of staff training have suggested these mean required training periods: (a) 6 h to correctly implement discrete trial teaching (Leaf, Aljohani, et al. 2018); (b) 2 h to correctly implement formal preference assessments (Leaf et al. 2019); (c) 3 h to correctly construct graphs (Leaf 2019), and (d) 4 h to correctly implement the teaching interaction procedure (Green et al. 2019). The average person, then, requires at least 15 h to learn just those four skills. Considering the 3 h ethics requirement (Behavior Analyst Certification Board 2018b; Behavior Analyst Certification Board 2020), the average person is left with only has 22 h (on average) to learn all of the other skills on the RBT® task list. Based upon previous research (e.g., Green et al. 2019, Leaf, Aljohani, et al. 2018), it seems unrealistic that 40 h is enough time to teach the entire RBT® task list items to mastery, let alone fluency (Binder 1996).

While the BACB® has not provided a rationale for the selection of 40 h as the requirement, they have stated this is a minimum requirement which trainers may exceed (Behavior Analyst Certification Board® n.d). However, there is no mechanism in place to determine if this occurs. It is also questionable as to whether funding agencies (e.g., stakeholders who have made the RBT® credential a requirement) would fund training hours that exceed minimum requirements specified by the BACB®. Unless longer duration of training is mandated, it is doubtful that most agencies will provide more than the minimum number of training hours.

### #3: Missing Skills on the Task List

Two important additions to the RBT® task list and competency-based assessment were shaping and token economies; however, their inclusion corresponded with the removal of other critical skills such as stimulus fading. The BACB® (Behavior Analyst Certification Board 2018b) stated that stimulus fading procedures were “removed due to concern of being too advanced for most entry-level RBTs” (p. 3). This removal is concerning because there does not appear to be any empirical investigations documenting that stimulus fading procedures are more difficult to learn or implement than other procedures. Further, there is research documenting their usefulness and effectiveness with the clients and situations in which RBTs® are most likely to work (e.g., Shabani and Fisher 2006).

A number of other potential critical learning objectives are also missing from the current RBT® task list. First, there are no learning objectives surrounding curriculum design or selection of curricular objectives. An understanding of curriculum design allows an RBT® to be responsive to the learner’s performance and problem solve within a session to establish the conditions under which a student demonstrates a skill (Leaf 2019). Effective, quality treatment requires constant assessment and analysis of a number of factors which the interventionist then uses to alter the curriculum as well as the teaching or behavior strategy in-the-moment. Without this skill, an RBT® may be required to wait for a supervisor to make curricular adjustments, which may not occur for extended periods of time and result in interrupted or halted progress.

Second, there are no learning objectives on the identification of evidence and non-evidence based procedures (Foxx in press). Implementing non-evidenced based procedures could undermine the effectiveness of an ABA program or turn the ABA program into an “eclectic model” (Foxx and Mulick 2016), which evidence does not support (e.g., Howard et al. 2014). Also, RBTs® typically have frequent contact with parents of the children with whom they work and are often among the most trusted team members for many families. Failure to support the development of this repertoire could result in an RBT® recommending and possibly implementing procedures with no empirical support, evidence base, or with pseudoscientific characteristics (e.g., fidget toys, weighted vests, or *Superflex*®*: A Superhero Social Thinking Curriculum*). Given that the BACB® receives complaints about BCBAs® implementing non-evidence based procedures (Bailey and Burch 2007), despite their training and certification, it is reasonable to expect the problem to be proportionally greater with an RBT® population that receives much less formal scientific training.

A third critical skill missing from the current RBT® task list is developing a therapeutic relationship in interactions with parents, teachers, and outside professionals (Callahan et al. 2019; Taylor et al. 2019). These fundamentally human relationship skills consist of, but are not limited to, displaying empathy, compassion, building rapport, and answering parents’ or professionals’ questions in a genuine and professional manner. If these skills are not a priority in the training of RBTs®, they may work less effectively with caregivers or other professionals (Taylor et al. 2019) and lead caregivers to perceive the field of behavior analysis as uncaring. As a result, parents might decide to abandon behavioral intervention in search of more “humane” (albeit less effective) approaches. There is also a need for some degree of skill related to cultural responsiveness to the child and family. That is, there is a growing understanding that the field of behavior analysis, as a whole, is not prepared to work with families of diverse cultural backgrounds with whom they have daily and influential contact (Beaulieu et al. 2018).

### #4: No Operational Definitions for Task List Items

Many of the items on the RBT® Task List require operational definitions due to a lack of clarity. For example, does “prompting and prompt fading” mean an RBT® has to be proficient in least-to-most prompting, most-to-least prompting, constant time delay, no–no prompt, flexible prompt fading, and/or simultaneous prompting? Are RBTs® required to have a generalized skill set or merely implement one type of prompting system to teach one specific skill? One cannot accurately evaluate whether or not competency has been met without an operational definition.

### #5: A Limited Competency Assessment

The added requirement that some skills must now be evaluated via direct observation is noteworthy and laudable. However, assessment via role-play and interview is still permitted for the majority of skills (i.e., 86%). Unfortunately, assessing a candidate’s skills with role-play and/or interview generates several concerns including, but not limited to, (a) interviews test knowledge and not performance, (b) role-plays can be arranged to make the candidate successful, (c) role-plays are conducted in a much simpler context than intervention in the natural environment, and (d) a possible failure of say-do correspondence. All of these outcomes could create a misleading or an inaccurate impression of how the candidate would perform in actual teaching and clinical contexts (see Leaf 2017, 2019 and Leaf, Leaf et al. 2017 for a complete description of the concerns of assessing competency with role-plays and interviews).

Allowing training and assessment processes to occur in a decontextualized setting are problematic for other reasons. One is that there is no evaluation of how the candidate responds and adjusts based on the effects, or lack thereof, of the procedures being used. The result is that the candidate’s behavior is focused on learning a set of rules or a chain of behaviors rather than learning the relation between their actions and the learner’s response. It is critical that an RBT® learn the conditions under which particular teaching techniques are best incorporated, how their behavior affects the learner’s behavior, and how to adjust their teaching in response the learner’s behavior. There are several potential undesired effects when these skills are omitted from behavior analytic training and assessment.

First, from a learning and performance vantage point, the RBTs® response chain is not acquired under the appropriate stimulus or reinforcer control. The client’s responding does not set the occasion for, reinforce, or produce variation in any of the task list procedural topographies. Second, behavior analysis is a discipline known for producing effective, meaningful change. A problem across all three areas of certification, but most concerning at the RBT® level, is related to in-the-moment treatment decisions needed in response to the client’s behavior. RBTs® have the most direct contact with clients which means their very important function is to produce behavior change by implementing an intervention, evaluating the client’s response, and quickly and appropriately responding to that response. It is conceivable that omitting this important skill could lead to us to train an entire generation of practitioners that clients’ responses to interventions are inconsequential.

### #6: Final Assessment Remaining a Multiple Choice Exam

A hallmark of ABA has been directly observing and evaluating behavior as opposed to a corollary of behavior (Baer et al. 1968, 1987). We recognize the premise that the direct observation of candidates for BCBA® and BCaBA® credentials may be impractical given the breadth and depth of skills to be assessed, as well as the logistical considerations. However, the limited scope and circumscribed activity (i.e., implementation of skills) makes direct performance of the target skills the only valid measurement of competence for the RBT® certificate. Nonetheless, the final assessment to become an RBT® remains an MCQ exam as opposed to a performance-based assessment. This is unfortunate because performance-based assessment meets our field’s credo that we care more about what persons do in a situation than what they say they will do (Baer et al. 1968, 1987; Gilbert 1978). It should be noted that while performance-based assessments are less common with regard to certification, there are certainly certifications in which performance based assessments have been used and accredited by the same accreditation body that has accredited the RBT® (i.e., the National Commission for Certifying Agencies 2014).

### #7: Potential for the Development of Conflicts of Interest and Dual Relationships

The concern for the potential for conflicts of interest and dual relationships remains. Although some safeguards are in place (e.g., a supervisee cannot be the employer of the supervisor; Behavior Analyst Certification Board 2018a), other important contingences leading to dual relationships and conflicts of interest have not been addressed. Most notably, with funding sources beginning to require certification for reimbursement, employers have a strong financial incentive for their staff to become certified as quickly as possible. When employers and evaluators’ livelihood depend on candidates passing, there is greater risk of bias in their determination of what constitutes competency.

### #8: The Subject Matter Expert (SME) Process

Currently, the BACB® allows individuals to submit a form on their website to volunteer as an SME if they meet specific qualifications. The BACB® reviews applications and determines SME assignments based on demographics, education/training, employment, experience, and geography. Although this process represents rules and regulations established by the National Commission of Certifying Agencies, it does not mitigate concerns. For example, the BACB® board of directors makes the final determinations regarding who serves as an SME on committees and panels, which may result in potential biases toward a certain educational lineage and/or dominant treatment/training approach within the field. Thus, it is possible that the board could select only SMEs who share a specific vision for certification that the board members value [e.g., favoring approaches expanded from the UCLA Young Autism Project (Lovaas 1987), those grounded in Skinner’s (1957) *Verbal Behavior*, or those excluding respondent conditioning]. Currently there is no transparency as to how many have applied to be an SME and the nature of any inclusion and exclusion criteria employed by the BACB®. Thus, it is impossible to determine how representative SMEs are of the field as a whole or if any systematic biases exist. Indeed, even though the BACB® board is elected by its certificants, the potential for biases remains. Consider that to participate in the voting, current certificants are required to request access to the ballot through the BACB®. The procedural constraints regarding the selection of SMEs and election of board members present problems with restricted representation and voting and selection practices that are non-transparent, which could result in biases that restrict the task list and certification such that they are not wholly representative of the field.

There is another concern regarding the SMEs and the decisions they make. Based upon the BACB® December 2018 Newsletter, a committee of 12 SMEs from different geographical regions, histories, and certifications (i.e., RBT®, BCBA®, BCBA-D®, BCaBA®) was convened. The newsletter also stated that a second panel comprised of six individuals, primarily RBTs®, also met in 2018. Although this description provides some important information, it raises other questions (e.g., Were the majority of the SMEs on the committee RBTs®, BCaBAs®, BCBAs® or BCBA-Ds®?; Were any of the SMEs consumers of behavioral intervention or members of professions outside of behavior analysis or behavior analysts who did not hold certification at any level?). If the SMEs focusing on RBT® certification were largely comprised of BCaBAs® or RBTs®, then the future of our discipline and decisions regarding important skills to include in these certification requirements is being decided by professionals in our discipline with the least amount of required training.

### #9: Ethical Concerns

The new RBT® standards also include a newly developed ethical code that includes 31 standards across three broad domains (i.e., responsible conduct, responsibility to clients, and competence and service delivery; Behavior Analyst Certification Board 2018a). The addition of an ethical code for RBTs® is important given anyone providing intervention for individuals diagnosed with ASD must adhere to ethical standards to ensure the safety and welfare of the clients with whom they work. However, the RBT® ethical code does not include some standards that are in the ethical code for BCBAs® and BCaBAs® that are applicable to RBTs® as well. For example, the RBT® ethics code does not include reliance on scientific knowledge. It seems necessary for those most responsible for providing direct intervention should be held to the same scientific standards as supervisors. The RBT® ethics code also provides no statement regarding effective treatment. While one may argue that is the responsibility of the supervising BCBA®, given the sometimes infrequent interaction with supervisors in comparison to treatment hours it seems appropriate that RBTs® should also be bound by a similar requirement. Given the number of ethical violations reported, investigated, and substantiated each year for BCBAs® and BCaBAs® (Behavior Analyst Certification Board 2018c), particularly those related to supervision practices, it seems risky to not expand the ethical code for RBTs® to include many of the areas included on the ethical code for BCBAs® and BCaBAs®.

### #10: Limited Research on the RBT® Certification

Our final concern is the paucity of research on the RBT® certification in general, and, more specifically, on best practices for training RBTs® who provide intervention for individuals diagnosed with ASD (Leaf, Leaf et al. 2017). In one of the few examples of research related to the RBT® credential, Fisher et al. (2014) used a randomized control trial to evaluate a 40 h virtual training program for eight participants eligible for the RBT® credential. Fisher et al. evaluated the effects of the training program on the participants’ demonstration of tasks found on the RBT® task list when implemented with a confederate serving as a client. There were significant differences between participants assigned to the group that received training compared to the group that did not. Although it can be concluded that trained participants’ skill level increased, it was not apparent if they were proficient in the improved skills. Additionally, participants did not display skills with clients, making it unknown if the skills generalized to the terminal context or if the acquired skills produced behavior change for individuals diagnosed with ASD.

Forte et al. (2018) evaluated the training process outlined by the BACB® with nine participants eligible for the RBT® credential. The training consisted of either role-play or in vivo training on how to implement discrete trial teaching, error correction, and reinforcement procedures. Forte and colleagues measured participant demonstration of the targeted skills with a confederate as well as generalization to actual clients. The results indicated that the participants could demonstrate the skills with confederates, but the skills did not maintain over time. Furthermore, and most relevant to our concern the skills did not generalize to clients.

To our knowledge there is no study that demonstrated significant behavioral outcomes for clients when intervention is conducted by RBTs®. That is, there is nothing at this time to suggest that RBT® involvement in treatment actually results in delivery of high quality intervention resulting in socially and clinically significant outcomes. Collecting outcome measures for recipients of services after staff members have completed 40 h trainings based on the RBT® task list could provide such information. The lack of empirical data showing a relationship between RBT® certification and learner outcomes begs the question of whether the field should adopt such a certification, given that the field of ABA has always been considered a scientific discipline.

## Potential Solutions

What follows are suggestions for potential solutions related to the aforementioned concerns. It should be noted that many of the concerns are related to complex systems and contingencies, as a result, some of the following solutions are complex, long-term, and, some may be considered, aspirational. It is likely there are other solutions we have not considered or developed. We encourage readers to consider the potential pragmatic challenges surrounding our proposed solutions and develop and share their own solutions.

### #1: Develop Clear Criteria for becoming Assistant Trainers and Competency Assessors

Clear and objective criteria for trainer skills should be specified. Trainers could be required to take a course on supervision, have an increase in the required number of supervision hours, pass a performance-based exam directly related to supervision, and accumulate a minimum number of hours of experience delivering intervention. Additionally, the BACB® should specify that those holding only the RBT® credential should be excluded from being a trainer or assistant trainer. This change would be consistent with the decision of the BACB® in 2016 to remove “assisting with training stakeholders” from the RBT® task list (Behavior Analyst Certification Board 2016, p. 2) “due to concern about RBTs being permitted, or incorrectly perceived as permitted, to train others” (Behavior Analyst Certification Board 2018b p. 3). Given that the BACB® has determined that RBTs® should not be training stakeholders, it follows that RBTs® should not provide training to other potential RBTs®.

### #2: Increase and Align Minimum Training Hours with the Research

There are at least two potential, though not mutually exclusive, ways to ensure that the number of required training hours for the RBT® candidates aligns more closely with the empirical literature. First, the BACB® should create a task force that includes behavior analysts and other professionals (e.g., psychologists, educators) who are representative of several behavior analytic university programs, behaviorally based training programs, and treatment providers. The task force should evaluate each learning objective on the RBT® task list by conducting a review/meta-analysis of the current research on each objective. The task force could then determine the average number of hours of training time required for each objective, and provide the total average time across all task list objectives. Based on this analysis, the number of required training hours for the RBT® credential would be adjusted to align with the empirical literature. This would not displace the SME process; instead, it would complement and strengthen the SMEs’ recommendations and decisions.

A second, similar direction includes a collaborative effort for conducting experimental studies evaluating training effectiveness and efficiency. Researchers could evaluate the average duration required to train candidates to competently and fluently demonstrate all of the learning objectives on the RBT® task list. One way to do so is provide participants with 40 h of training on all items on the task list. After the 40 h, the researchers could provide an assessment of all items on the task list. If a participant does not reach a predetermined mastery criterion for any specific learning objectives on the task list, then additional training could occur until mastery was achieved. Such an evaluation conducted across a considerable number of participants in different settings would provide the BACB® with the objective data to establish training hour requirements based on empirical demonstrations rather than an arbitrary standard (i.e., 40 h).

It could be argued that increasing the minimum number of training hours might limit access to services or that funding agencies may not pay for increased training. That said, as a science-based discipline, the determination of the minimum number of hours of training someone needs to be certified to work directly with individuals diagnosed with ASD should be data based. We need research identifying the parameters of what constitutes necessary and sufficient training. In the interim, we should adhere to what we know about effective instruction (e.g., Binder 1996; Engelmann and Carnine 1982; Heward et al. 2005; Lindsley 1992; Skinner 1968; Tiemann and Markle 1978) and developing competent performance (Gilbert 1978).

### #3: Determine Skills to be Included on the Task List and Update Accordingly

To begin, the BACB® should reinstate learning objectives, such as stimulus fading, that have been removed from the RBT® task list. Second, the BACB® should attempt to determine and include all skills an RBT® needs to successfully work with individuals diagnosed with ASD. Instead of solely relying on SMEs, additional steps could be taken to ensure a more thorough process. To start, the BACB® should send out a survey to all BCBAs® working in the field of autism asking which skills are necessary for RBTs® to be successful. Additionally, SMEs should seek to identify learning objectives by reviewing curricular books and empirical research and interviewing agencies known to deliver quality interventions and services. This confluence of actions would go a long way in the creation of a more exhaustive list of skills an RBT® needs to be successful. Third, it should be required that RBTs® receive training related to developing therapeutic relationship skills and cultural responsiveness. Although this would likely necessitate an increase in the total required training time, the net result should be improved treatment outcomes, stakeholder satisfaction, and collaboration. This work has already started. For instance, Taylor et al. (2019) have outlined a potential curriculum for building these skills and other investigators have included relationship building and rapport building components in their intervention and training programs (e.g., Blell et al. 2010; Weinkauf et al. 2011; Willner et al. 1977).

### #4 Develop Operational Definitions for Task List Objectives

The BACB® should provide operational definitions or, at the very least, parameters and context for each of the task list objectives. This would be done by examining the definitions used in empirical research and curricular books. If an operational definition cannot be found, SMEs could create one. This change might also serve to improve the consistency of evaluations conducted across different RBTs® in various settings and limit the risk of subjectivity by assessors who administer RBT® evaluations. We would also like to note that in an effort to avoid undue rigidity while clarifying task list objectives, lists of examples and non-examples may be useful. We recognize that defining these skills clearly will almost certainly result in a need for more time to teach the breadth and depth of the skills and that this may be a possible explanation for their current absence. Nevertheless, our position is that the quality of behavior analytic services delivered should always supersede what may appear to be a logistical or pragmatic concern (i.e., the time required or allotted for training and instruction).

### #5: Require Competency-Based Assessment Exclusively Within the Terminal Context

The solution here is already partially found within the RBT® Competency Assessment Requirements (Behavior Analyst Certification Board® n.d). That is, an RBT® candidate is required to perform each task on the task list while being directly observed by the assessor. However, under the current process, this performance may occur with a client or through role-play. Given recent research findings (e.g., Forte et al. 2018), there should be no expectation that skills performed in a role-play will generalize to clients. To solve this problem, the BACB® should require that competency-based assessments be conducted exclusively in the actual environment in which the prospective RBT® will perform the skills on the task list. An ethical and logical exception would be assessment of the RBT® implementing crisis/emergency procedures through role-play.

### #6: Evaluate a Greater Portion of the Task List Skills in a Performance-Based Format

Assessing only three skills with a client appears to be arbitrary. We ask that the BACB® require evaluating a greater portion of the task list skills in a performance-based format. Skills such as discrete trial teaching, shaping, or incidental teaching, for example, should be considered for a performance-based assessment, not just a competency-based assessment. Additionally, the method of evaluation should include direct observation with a criterion referenced checklist. Alternatively, candidates could submit a videotape of themselves demonstrating the skill to the BACB®, and have two or more qualified professionals score the videotape using pre-established criteria. This would be similar to certification on the Autism Diagnostic Observation Scale (ADOS; Lord et al. 2012), a gold standard instrument in assessment.

We recognize there are potential barriers related to a performance-based assessment such as cost, time, logistics, and defensibility (Carr et al. 2017). While a performance-based assessment is more costly than an MCQ examination, it clearly represents a more valid assessment of the skills that RBT® credentialed individuals are expected to perform. Ensuring candidates can actually perform the required skills should result in higher quality intervention, which should result in better outcomes and lower costs of treatment and care over the lifetime of the treated individual (e.g., Koegel et al. 2014).

Another argument against performance-based assessments is increased difficulty for the legal defensibility of learning objectives, which make it difficult to align with best practices in the credentialing industry. Nevertheless, the field of ABA has long been concerned with the precision of objective measurement (Baer et al. 1968, 1987; Lindsley 1992). Researchers have been able to operationally define complex skills like creativity (e.g., Goetz and Baer 1973), problem solving (e.g., Foxx and Faw 2000), social engagement (e.g., Shireman et al. 2016), and friendship (e.g., Hanley et al. 2007). Given these operationally defined advanced skills and skill sets and the plethora of studies containing operational definitions, we are confident that members of the BACB® and SMEs can operationally define the basic tasks that an RBT® should display. These definitions should result in criteria that are legally defensible.

Although performance-based assessments are less common with regard to certification, there are certainly certifications in which performance based assessments have been used (e.g., Certified Culinarian©, Certified Sous Chef©, Certified Executive Chef© by the American Culinary Federation; operate a rigger, lattice boom, or digger derrick by the Crane Institute Certification; operate an overhead crane by the Operating Engineers Certification Program) and accredited by accreditation bodies (e.g., the American National Standards Institute 2016; the Institute for Credentialing Excellence 2016; the National Commission for Certifying Agencies 2014), the same governing bodies that have accredited the RBT®. While these examples may not relate directly to behavior analysis, they were selected purposely to highlight that professions can include performance-based assessments while achieving accreditation from the same bodies that accredit behavior analytic certifications (i.e., National Commission for Certifying Agencies 2014).

Unfortunately, professionals have questioned the effectiveness of behavioral intervention for individuals diagnosed with ASD (Kuperferstein 2018) despite overwhelming evidence to the contrary (see Foxx 2016). Interventionists who are not competently implementing behavioral intervention could lend credence to these critiques. If our certification process does not ensure competency it can have a serious impact on the field of behavior analysis and the welfare and long-term outcomes of our clients. The onus is on our field to strive to use the most valid and stringent certification assessment possible, which is why we believe that performance-based assessment is critical.

### #7: Use Independent Assessors for Competency-Based Assessments

Agencies and professionals susceptible to potential conflicts of interest related to financial contingencies should not conduct assessments for certification. Simply put, no agency that will receive remuneration for services rendered by that RBT® candidate should conduct assessments leading to that person’s certification. In fact, the person conducting certification assessments should have no financial gain or loss as a result of the candidate passing or failing. Companies and professionals who need their employees certified should seek to build collaborations with independent assessors.

### #8: More Transparency Related to the SME Process

There should be more transparency related to who is functioning as an SME and to which projects each is assigned. Doing so provides the behavior analytic community with assurances that no potential biases occur within this important contribution to the certification processes. Second, there should be more transparency in the inclusion and exclusion criteria used to select SMEs. It is important that these criteria include evaluations of the SMEs’ practice and scholarship experience. Third, SME committees and panels should include non-certified individuals (e.g., parents, teachers, other professionals including psychologists) who come into contact with RBTs®.

### #9: Expand the Current RBT® Ethical Code

The BACB® should expand its current ethical code to address ethical dilemmas unique to an RBTs® as a result of their frequent, direct contact with clients and families. A starting point could be including the relevant contents of the BCBA® and BCaBA® Professional and Ethical Compliance Code for Behavior Analysts (Behavior Analyst Certification Board 2018a). For example, the RBT® ethical code should also include a section related to a reliance on scientific knowledge. The ethical code for RBTs® more closely resembling the Professional and Ethical Compliance Code for Behavior Analysts (Behavior Analyst Certification Board 2018a) would provide, at a bare minimum (though far from ideal), a set of rules and standards for RBTs® to follow (Rosenberg and Schwartz 2019).

### #10: Promote Research Related to the RBT® and Align Standards with that Research

The RBT® certification has clearly become part of the field, and we expect that periodic review of the credentialing standards and training requirements will continue. However, it is essential that future changes to certification training requirements and standards be guided by evidence. Simply stated, there is currently not sufficient data to suggest that the RBT® credential actually results in delivery of high quality intervention resulting in socially and clinically significant outcomes. There needs to be more research on the RBT® training, assessment, certification, and associated outcomes.

Research is required that evaluates how many hours of RBT® training are needed and what instructional strategies should be used to produce proficiency across all required skills. Initial studies could be descriptive. For instance, agencies could collect and report data on the number of hours of training required for staff to demonstrate proficiency on each task list item. Single-subject research methodology could then be used to evaluate individual participant demographics and training modalities that affect the number of hours required for proficiency to be demonstrated on the various task list items. Between-group design studies could also be used to evaluate the average number of hours required across populations and settings for proficiency to be demonstrated on all the task list items.

There also needs to be research conducted on best practice in training RBTs® to reach proficiency. Single-subject research methodology has already been used to evaluate the effectiveness of various training procedures (e.g., Higbee et al. 2016). As this research base grows, comparative studies could be used to evaluate the relative effectiveness of two or more training procedures used to teach different items from the task list. Long-term data regarding the intervention outcomes produced by RBTs® are also needed. Specifically, research must be conducted that evaluates the comparative effectiveness of intervention programs implemented by those with an RBT® credential, without the credential, and those with higher-level credentials, controlling for variables such as level of education, level or degree of mentored experience, and experience more generally defined. These comparative studies could provide valuable information to guide future decision making related to certification standards and training.

## Discussion

Twenty members of the field of behavior analysis have collectively contributed to this commentary because we are concerned about the impact of the RBT® credential on our field. We acknowledge this as a continued professional discussion begun by Leaf, Leaf et al. (2017) and Carr et al. (2017). As previously noted, our concerns stemmed, in part, from RBT® requirements determined by means other than objective data, and revisions curtailing training requirements. Research is required demonstrating the RBT® credential correlates with significant outcomes to prevent the potential for undesired consequences (e.g., providing funding for less effective services that have less or no empirical support). Failing to take action to prevent these undesired consequences could be tragic for the families and clients we serve if they result in losing the opportunity to achieve best outcome, failing to achieve best outcome, and, in many cases, experiencing worse outcomes.

We are currently in a culture in which the appropriateness of behavioral intervention for individuals diagnosed with ASD is being questioned, including such drastic claims that it causes symptoms of PTSD (Kupferstein 2018; but also see Leaf, Ross, et al. 2018). It is a time during which an increasing number of non-behavioral interventions are recommended for individuals diagnosed with ASD (Lilienfeld et al., 2015), some of which have some evidence (e.g., Early Start Denver Model) while many are pseudoscientific (e.g., Social Thinking®). A variety of other challenges related to non-behavioral interventions for individuals with autism are discussed and refuted in Foxx and Mulick (2016). The increase in pay-to-publish journals presents an additional challenge, making it difficult for lay persons to know what is and is not quality, peer-reviewed research. Perhaps the greatest challenge to our field concerns funding. It is conceivable that funding agencies could become less supportive as a result of reports that ABA services produce negligible outcomes, despite there not being any demonstration of experimental rigor or control.

The concerns and solutions offered here relate to the initial training related to obtaining the RBT® credential. There may be other solutions that could be beneficial that would occur after RBT® certification is obtained. For example, individuals holding an RBT® certification could be required to complete ongoing continuing education to maintain the certification. This would more closely align with other behavior analytic credentials (e.g., BCBA®, BCaBA®). It should be noted, however, the BACB® already requires RBTs® receive ongoing supervision and renew/complete a competency assessment each year to maintain their certification (Behavior Analyst Certification Board 2020). Additionally, the BACB® has developed an auditing process for RBTs® (Behavior Analyst Certification Board, 2020). The BACB® should be commended for both of these efforts to help ensure maintenance of the skills an RBT® is required to display.

Ultimately, this is a time with great potential, but also great uncertainty. It is time for our discipline to critically evaluate our certification standards and the resulting quality of outcomes for individuals diagnosed with ASD. The authors of this manuscript believe the current standards of RBT® certification are in need of revision and have provided ambitious solutions in an effort to start the process. These solutions will most likely result in an increase in the amount of training time required for RBT® candidates, the training costs for service providers and agencies, and, on a global level, make it more difficult to scale behavioral intervention. However, we believe that these solutions are necessary to ensure continual improvement in behavioral interventions and outcomes for individuals diagnosed with ASD.

## Footnotes

1 Although not all certified behavior analysts provide direct intervention or work in the field of autism intervention, recent numbers suggest the majority of certified behavior analysts do work within autism and autism intervention (Behavior Analyst Certification Board® n.d.).

2 The RBT® credential, perhaps, more closely aligns to those who provide direct intervention for individuals diagnosed with ASD.
